# Effects of age and speed on the ankle–foot system’s power during walking

**DOI:** 10.1038/s41598-020-71763-8

**Published:** 2020-09-10

**Authors:** Lucas Santana da Silva, Reginaldo Kisho Fukuchi, Renato Naville Watanabe, Claudiane Arakaki Fukuchi, Marcos Duarte

**Affiliations:** 1 Biomedical Engineering Program, Federal University of ABC, Rua Arcturus, 3, São Bernardo do Campo, SP CEP 09606-070 Brazil; 2 Department of Orthopedics and Traumatology, Faculty of Medical Sciences, University of Campinas, São Paulo, Brazil

**Keywords:** Biomedical engineering, Skeletal muscle, Ageing

## Abstract

Structural and functional changes in the foot have been associated with age-related changes in gait mechanics, but walking speed may be a confounding factor in this relationship. The aim of this study was to investigate the effect of aging and speed on the ankle–foot power output during level walking. The effects of speed and aging on features of the mechanical power and work of the ankle and foot were quantified with a gait analysis of 24 young and 16 older individuals walking at different speeds. We observed gait speed having a significant effect on all the investigated features: peak power and positive and negative work of the ankle, foot, and sum of the ankle and foot (average effect size: 0.64 ± 0.22, from 0.26 to 0.87). We observed age having no effect on these same features (average effect size: 0.23 ± 0.12, from 0.03 to 0.39), with the exception of age’s effect when combined with speed on the negative work of the foot. We performed additional analysis to illustrate how the speed can become a confounding factor to the understanding of the age effect on the gait biomechanics. Based on the influence of gait speed on the mechanical power of the ankle–foot system, it is essential that studies control for the effect of gait speed if there is interest in understanding age-related effects, particularly when studying frail older individuals.

## Introduction

The human foot is literally and figuratively the basis for our upright standing and locomotion over a solid substrate. Structural and functional changes in the foot, including deformities^1^, increased soft tissue stiffness^2^, decreased range of motion^3^, and decreased strength^4^, have been associated with age-related changes in balance and gait, such as an increased risk of falls^5^, reduced joint mobility^6^, reduced gait speed^7^, and less efficient and stable walking^8^, ultimately worsening the quality of life of older individuals^9^. Aging is a progressive and gradual process, it affects people differently, but deleterious changes usually become more visible after 60 years of age^10^. Particularly, the decline in muscle strength with aging is more pronounced in the lower body than in the upper body^11,12^, and it has been suggested that aging is associated with a distal-to-proximal redistribution in joint torques and powers during walking, meaning that elderly individuals tend to use their hip extensors more and their ankle plantar flexors less than young adults^13^.

Slower gait speed is usually associated with aging and, considering the effects of speed on gait biomechanics, variations in walking speed, within the subject or between subjects, can confound the association between a demographic predictor, such as age, and a gait biomechanics feature^14–17^. In general, maximum amplitudes of lower limb joint kinetics are directly proportional to gait speed^14,17^. Of note, some studies have found that young and older individuals adopt similar gait speeds, at least while walking at their self-selected pace in laboratory settings^18,19^. On the ankle–foot system, a meta-analysis found a decrease in ankle kinetics with aging, with and without consideration of walking speeds^15^. When the analysis was restricted to studies with similar or matched walking speed, the meta-analysis found a significant overall standardized effect of age on ankle kinetics equal to − 0.85. Nevertheless, the literature is divided, exactly half of the 20 studies included in this speed-matched analysis did not find a significant age-related effect on ankle kinetics.

Another complicating factor for the investigation of gait mechanics concerns how the ankle–foot system is modeled: despite the fact that the foot is a complex structure with multiple segments, it has typically been modelled as a rigid body in gait studies, but the use of this approach has been shown to overestimate ankle–foot kinetics^20,21^. An alternative approach has been proposed to address these limitations: the ankle–foot system is modeled as a deformable segment, and there is consideration for the six degrees of freedom of the ankle joint^21–23^. This alternative approach not only yields a more complete description of the lower-extremity distal power profiles, but also allows for examining the power profiles of the ankle and foot separately. Considering all the previously discussed age-related alterations in the foot, investigating foot kinetics using such an approach, which is yet to be applied to study the gait of an older population, might contribute to a greater understanding of foot biomechanics during walking.

Therefore, the aim of this study was to investigate the association between aging and the ankle–foot power output considering the effect of gait speed. We hypothesized that (1) gait speed would affect foot–ankle power profiles during the stance phase of walking for both young and older adults and (2) once gait speed is controlled for, older adults would have similar foot–ankle power profiles compared to young adults.

## Results

**Figure 1:**
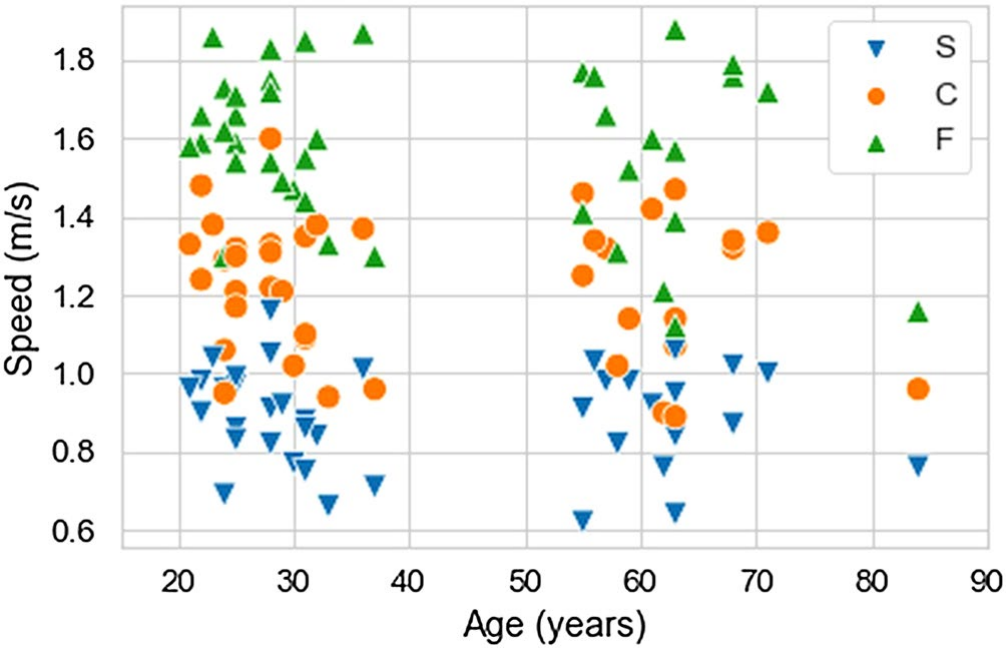
Subjects' walking speed versus age (N = 40). Walking speed conditions: slow (S), comfortable (C), fast (F).

Characteristics of the subjects and their walking speeds are presented in Table 1 and Fig. 1. The older individuals were significantly shorter, by 10 cm on average, than the young individuals, but the average leg length, which would have a greater effect on their gait biomechanics, was not different between groups. There was no difference in walking speed between the two age groups. Note in Fig. 1 that the subjects are clustered in two age groups, and even though the subjects were asked to walk at three different speeds, considering all subjects, the gait speeds are continuously spread over an interval. These findings influenced us to consider age as a categorical factor and speed as a continuous factor in our main analysis.

**Table 1:** Number of subjects and mean (± 1 standard deviation) of age, height, mass, BMI, leg length, walking speeds and stance time of the young and older subjects.

	Age group		Statistics
	Young (N = 24)	Older (N = 16)	(t, p, d)
Age (years)	27.6 ± 4.4	62.9 ± 7.4	16.5, < **0.001**, 5.74
Height (m)	1.71 ± 0.11	1.61 ± 0.10	− 3.02, **0.005**, 0.94
Mass (kg)	68.4 ± 12.2	65.8 ± 10.1	− 0.73, 0.472, 0.23
BMI (kg/m^2^)	23.4 ± 4.0	25.3 ± 3.7	1.56, 0.128, 0.50
Leg length (m)	0.88 ± 0.06	0.83 ± 0.07	− 1.93, 0.064, 0.65
Speed (m/s)			
Slow	0.88 ± 0.13	0.88 ± 0.13	0.19, 0.853, 0.06
Comfortable	1.23 ± 0.17	1.21 ± 0.20	− 0.24, 0.809, 0.08
Fast	1.60 ± 0.16	1.54 ± 0.24	− 0.82, 0.422, 0.29
Stance time (s)			
Slow	0.79 ± 0.08	0.77 ± 0.09	− 0.54, 0.596, 0.18
Comfortable	0.63 ± 0.05	0.61 ± 0.07	− 0.99, 0.330, 0.34
Fast	0.55 ± 0.05	0.54 ± 0.05	− 0.20, 0.845, 0.06

Also presented are statistics for differences between the groups using independent two-tailed *t* tests: t statistic (t), p value (p), and effect size (d). Significant differences are shown with p values in bold.

**Figure 2:**
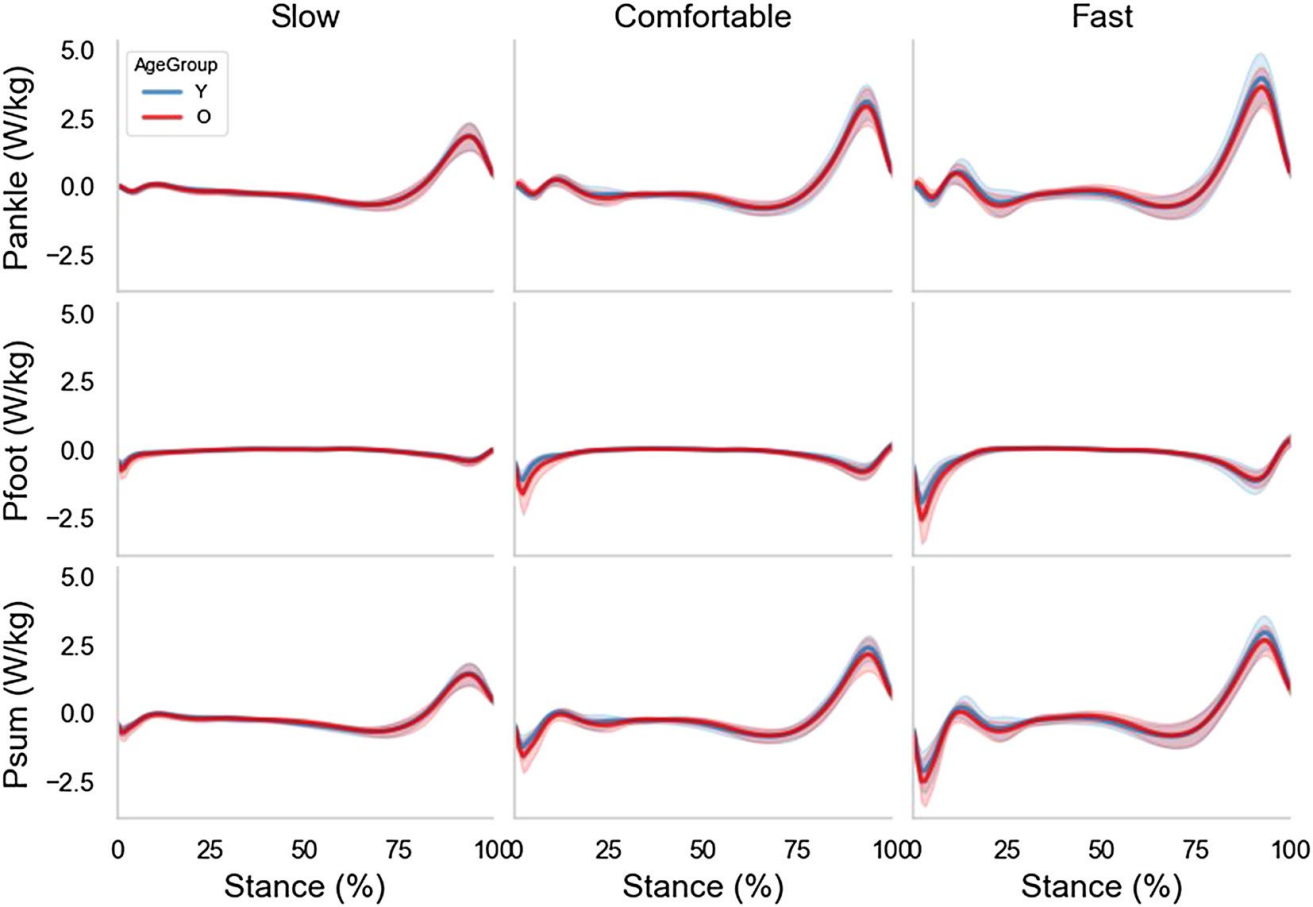
Time-series averages across subjects (± 1 standard deviation) for ankle power (Pankle), foot power (Pfoot), and sum of the ankle and foot powers (Psum) for the groups of young (N = 24) and older (N = 16) individuals during the stance phase of walking grouped by the subjects’ slow, comfortable, and fast speeds.

Figure 2 shows the average across-subjects time series of the ankle, foot, and sum of the ankle and foot powers for the young and older individuals during the stance phase of walking at the subjects’ slow, comfortable, and fast speeds. Overall, the power patterns are similar between the groups and among speeds, with an increase in the peak amplitudes as the speed increases, and consistent within the groups (the latter being indicated by the small standard deviations of the average patterns).

**Figure 3:**
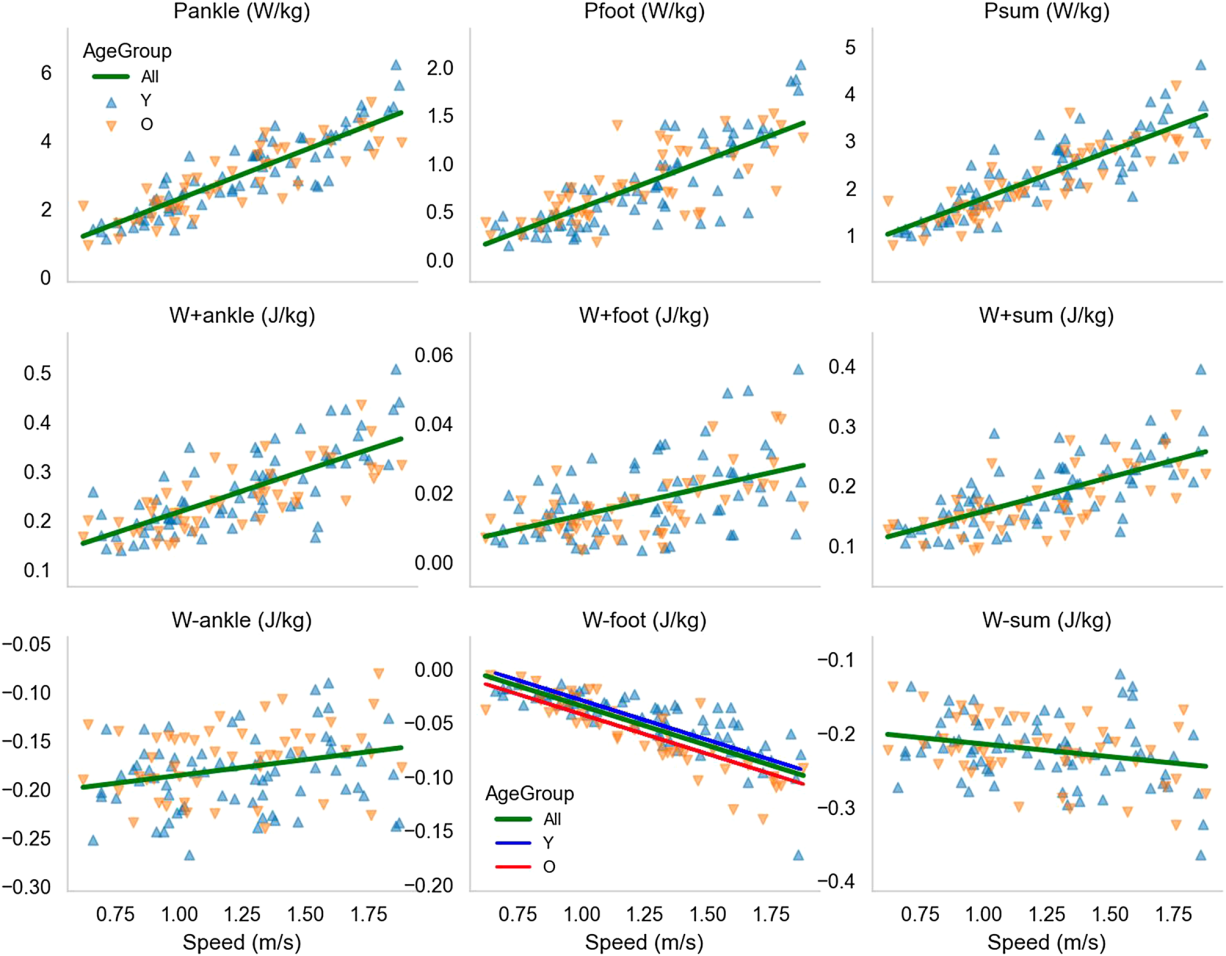
Scatter plots for the features peak power, total positive work, and total negative work of the ankle, foot, and sum of the ankle and foot (see plots’ subtitles) versus walking speed and corresponding fitted linear regressions with the model ‘feature ~ speed’ for all combined data (All) of the young (Y) and older (O) groups. Only for the W-foot feature, the model ‘feature ~ speed + AgeGroup’ also yielded significant effects and the corresponding linear regression for each AgeGroup is shown.

The results of the linear mixed-effects regressions revealed a significant effect of the Speed factor for all investigated features of the biomechanical variables without any effect of the AgeGroup factor with the exception for the total negative work of the foot (W-foot). For the W-foot feature, a significant effect was observed using the ‘Speed + AgeGroup’ and ‘Speed × AgeGroup’ models (but not AgeGroup alone), but with a small improvement in the model fit in relation to the model with the Speed factor alone (see Table 2 and Data availability). The plots for the statistically significant regressions of the investigated features versus the Speed factor are presented in Fig. 3.

**Table 2:** Results of the linear regression models having as possible predictors (in blocks of rows): only Speed (first row), only AgeGroup (second row), Speed + AgeGroup (third row), and Speed + AgeGroup + Speed × AgeGroup (fourth row), for each of the features: peak power (P), total positive work (W+), and total negative work (W−) at the ankle, foot, and sum of the ankle and foot.

Feature	Predictor						Goodness of fit		
	Speed		Age		Speed × Age				
	β_S_	CI	β_A_	CI	β_SA_	CI	LLF	AIC	R^2^
Pankle	0.87**	[0.80, 0.93]					− 66.8	141.6	0.78
			− 0.19	[− 0.57, 0.19]			− 171.4	350.9	0.01
	0.87**	[0.80, 0.93]	− 0.11	[− 0.37, 0.14]			− 67.5	145.1	0.78
	0.91**	[0.83, 0.99]	− 0.12	[− 0.37, 0.14]	− 0.12	[− 0.25, 0.00]	− 67.6	147.2	0.78
Pfoot	0.79**	[0.70, 0.88]					− 102.8	213.5	0.63
			− 0.03	[− 0.43, 0.36]			− 171.6	351.2	0.00
	0.79**	[0.70, 0.88]	0.04	[− 0.28, 0.35]			− 103.7	217.3	0.63
	0.85**	[0.73, 0.96]	0.03	[− 0.27, 0.34]	− 0.15	[− 0.33, 0.03]	− 103.8	219.6	0.64
Psum	0.83**	[0.77, 0.90]					− 73.9	155.8	0.72
			− 0.26	[− 0.65, 0.14]			− 170.7	349.4	0.02
	0.83**	[0.77, 0.90]	− 0.18	[− 0.47, 0.11]			− 74.1	158.3	0.73
	0.88**	[0.80, 0.96]	− 0.18	[− 0.47, 0.10]	− 0.11	[− 0.24, 0.02]	− 74.5	161.1	0.73
W+ ankle	0.74**	[0.66, 0.82]					− 100.5	209.0	0.56
			− 0.25	[− 0.68, 0.19]			− 168.7	345.5	0.01
	0.73**	[0.65, 0.81]	− 0.18	[− 0.55, 0.19]			− 100.8	211.6	0.57
	0.79**	[0.69, 0.89]	− 0.18	[− 0.55, 0.18]	− 0.14	[− 0.30, 0.03]	− 101.0	213.9	0.57
W+ foot	0.52**	[0.40, 0.64]					− 141.7	291.3	0.27
			− 0.21	[− 0.68, 0.26]			− 166.3	340.6	0.01
	0.52**	[0.40, 0.64]	− 0.16	[− 0.61, 0.28]			− 142.0	294.0	0.28
	0.47**	[0.32, 0.62]	− 0.16	[− 0.61, 0.29]	0.13	[− 0.12, 0.38]	− 142.6	297.2	0.27
W+ sum	0.66**	[0.58, 0.75]					− 109.1	226.2	0.49
			− 0.36	[− 0.82, 0.10]			− 165.2	338.4	0.03
	0.66**	[0.58, 0.75]	− 0.30	[− 0.69, 0.09]			− 108.7	227.4	0.51
	0.70**	[0.60, 0.81]	− 0.30	[− 0.69, 0.09]	− 0.11	[− 0.29, 0.06]	− 109.4	230.7	0.51
W-ankle	0.29**	[0.18, 0.39]					− 140.8	289.6	0.05
			0.39	[− 0.14, 0.93]			− 149.6	307.1	0.04
	0.29**	[0.18, 0.40]	0.42	[− 0.13, 0.97]			− 140.0	290.1	0.09
	0.26*	[0.12, 0.40]	0.42	[− 0.13, 0.97]	0.08	[− 0.15, 0.30]	− 141.1	294.1	0.09
W-foot	− 0.82**	[− 0.90, − 0.73]					− 98.2	204.3	0.65
			− 0.36	[− 0.73, 0.00]			− 170.1	348.2	0.03
	− 0.82**	[− 0.90, − 0.74]	− 0.44*	[− 0.71, − 0.16]			− 94.8	199.6	0.70
	− 0.72**	[− 0.82, − 0.62]	− 0.44*	[− 0.71, − 0.17]	− 0.26*	[− 0.43, − 0.10]	− 91.7	195.4	0.72
W-sum	− 0.26**	[− 0.37, − 0.15]					− 142.3	292.5	0.10
			0.04	[− 0.51, 0.60]			− 149.9	307.9	0.00
	− 0.26**	[− 0.37, − 0.14]	0.02	[− 0.52, 0.56]			− 142.7	295.3	0.10
	− 0.23*	[− 0.38, − 0.09]	0.02	[− 0.52, 0.55]	− 0.07	[− 0.31, 0.17]	− 143.7	299.4	0.10

The following statistics are presented: regression coefficients (slopes) for the terms Speed (β_S_), AgeGroup (β_A_) and Speed × AgeGroup (β_SA_), 95% confidence interval for the slopes (CI), log-likelihood (LLF), Akaike Information Criterion (AIC), and coefficient of determination between predicted and actual feature values (R^2^). Statistically significant (α = 0.05) slopes are in bold and the p value for the *t* test of the null-hypothesis significance test is indicated with * if p < 0.005 or ** if p < 0.0001. The coefficients for Speed (β_S_) and values of the features are standardized; AgeGroup is a categorical factor (Young = 0, Older = 1).

To verify the robustness of the results, we also ran the regression models treating age as a continuous variable and we obtained similar results as before: effects of speed on all features (average-across-features effect size: 0.64 ± 0.23, ranging from 0.27 to 0.87) and an effect of the models with the combined speed and age factors on the negative work of the foot. In addition, we observed a small effect of age on the W+ sum feature, but the R^2^ statistics of this model was only 0.06. See Data availability for the complete results.

Finally, we performed an analysis comparing only a subset with the slower half of older subjects (N = 8) and the faster half of young subjects (N = 12). As expected, the comfortable speed of the Young subgroup was significantly faster than the comfortable speed of the Older subgroup (Young = 1.37 ± 0.09 m/s, Older = 1.05 ± 0.13 m/s; CI = [− 0.44 − 0.21], d = 3.1). When we employed a linear regression model with the factor age alone (model ‘feature ~ AgeGroup’), we found that age yielded significant effects in six out of the nine investigated features (average-across-features effect size: 0.72 ± 0.21, ranging from 0.37 to 1.01). When we added the speed factor to the regression analysis (model ‘feature ~ AgeGroup + Speed’), speed affected all features (average-across-features effect size: 0.67 ± 0.26, ranging from 0.17 to 0.89) and the effect of age was nullified (average-across-features effect size: 0.31 ± 0.19, ranging from 0.13 to 0.73). See Data availability for the complete results.

## Discussion

The effects of speed and aging on features of the mechanical power and work of the ankle and foot were quantified with a gait analysis of the young and older healthy individuals walking at different speeds. In agreement with our first hypothesis, we observed gait speed having a significant effect on all the investigated features of peak power and positive and negative work of the ankle, foot, and sum of the ankle and foot (average-across-features effect size: 0.64 ± 0.22, ranging from 0.26 to 0.87). In agreement with our second hypothesis, we observed age having no effect on the majority of gait features (average-across-features effect size: 0.23 ± 0.12, ranging from 0.03 to 0.39), with an exception for the negative work of the foot (W-foot feature). For the W-foot feature, the models ‘Speed + AgeGroup’ and the interaction ‘Speed + AgeGroup + Speed × AgeGroup’ (but not AgeGroup alone) yielded a significant effect, but with a small improvement in the model fit in relation to the model with the Speed factor alone. We employed a method that considers the ankle and foot separately, and although this method has been applied elsewhere^21^, this is the first study to apply it to compare the ankle–foot mechanical power considering the effects of both gait speed and aging.

The finding in this study that walking speed affected all gait features is in agreement with the literature that generally states that increased walking speed demands increased power and mechanical work output in the lower-extremity joints^24,25^. In particular, the effect of walking speed on foot mechanical power output is far less understood compared to more proximal joints. W-foot was the only feature that presented a significant effect of the speed factor alone or combined with the age factor. This finding is highlighted in the ensemble time series curves displayed in Fig. 2, where older adults seem to absorb more power during the early stages of the stance phase compared to young people. In agreement with part of our findings, an increase in negative foot work when speed was manipulated in young subjects has been reported in both walking^26^ and running studies^27^. To our knowledge, no studies have examined the combined effects of speed and age on foot energetic profiles. However, it has been suggested that the foot and shoe are responsible for as much as 60–70% of the soft tissue work during early stance of level walking^28^ and a loss of elasticity in the heel pad with aging has been observed^29^. Hence, the observed age-related effect on the negative work of the foot during walking might be related to the deterioration of the heel pad property with aging, but this needs to be further investigated.

There has been a raising concern about the potential confounding effect of speed on the relationship between aging and gait biomechanics^14^. However, we didn’t find any difference between the gait speed of the two age groups, and therefore speed was not a confounding factor to the observed gait biomechanics, and also because we didn’t find any effect of age (with the exception on the negative work of the foot as discussed previously). Despite the fact the literature typically postulate that aging slows gait this has not always been the case^20,21^. To demonstrate a possible confounding effect of speed on age studies, we performed an analysis comparing only a subset with the slower half of older subjects and the faster half of young subjects (see Data availability). A regression analysis with the factor age alone yielded significant effects in most of the investigated features (as most studies have reported), but when the speed factor was added to the regression model, the effect of age was nullified and we obtained once again the same effects of speed on the investigated features, that is, revealing speed as a confounding factor.

Another factor that could have played a role in explaining age’s lack of effects on the features is the method used to calculate the ankle–foot power, as the majority of the studies cited in the meta-analysis did not consider both the power of the foot and the translational power at the ankle^15^. However, in the present investigation, neither Pankle nor Wankle, which were calculated similarly to previous studies, presented significant effects related to age; therefore, this finding strengthens the control of gait speed as the most determinant factor to explain the results.

The present study certainly has limitations to be acknowledged. The present study only included healthy young (18–40 years) and older (55 years and over) subjects; thus, the present results are only applicable to similar populations. Another limitation is that the foot segment was modelled as a single rigid body and thus the foot power could be overestimated by this simplistic approach, presumably due to the overestimation of foot angular velocity. We were aware about more appropriate models^21^ by the time this study was conducted but we were unaware when the data of the original data set study was collected.

We conclude that walking speed significantly affects both the ankle and foot mechanical power output profiles. In contrast, age, both in isolation or combined with walking speed, did not affect either ankle or foot power profiles, with the exception of age’s small effect when combined with speed on the negative work of the foot. Although the effects of the speed factor and its dominance over the age factor on gait biomechanics reported here are clear, we recognize that this topic still needs further investigation. At least because of the variability in motor behavior and the diversity in the elderly population. Finally, it is essential that studies control for the effect of gait speed if there is interest in understanding age-related effects, particularly, in studies where frail older individuals, who tend to walk slower, are considered.

## Methods

The gait data in this study were from an open dataset previously published elsewhere^30^. Forty healthy individuals from the open dataset with 42 subjects were included in this study and were assigned to the Young group (between 18 and 40 years old) or to the Older group (at least 55 years old). The subjects had to be free from any lower-extremity injuries in the past 6 months and not presenting neurological or orthopedic conditions that affected their gait pattern by the time the study was conducted. Further details about the participant selection can be found in the original study, which had each participant read and signed a consent form that had previously been approved by the ethics committee of the Federal University of ABC prior to the experiment (CAAE: 53063315.7.0000.5594) and where all experiments were performed in accordance with relevant guidelines and regulations^30^. From the 42 subjects of the open dataset, we excluded a total of two subjects; one because of the age group criterion, and one because of unacceptable force signals quality. For the included subjects, demographic and anthropometric information are presented in Table 1 and plots of their age and walking speed values are presented in Fig. 1.

The participants were required to perform level overground barefoot walking trials, first at their self-selected comfortable speed, and then at speeds 30% faster and 30% slower than the comfortable speed. A minimum of six representative trials were considered for each subject at each condition. These trials were manually selected using visual inspection of the ground reaction force and kinematic data to guarantee a trial where the subject stepped on the same force plate with only one foot at a time. Twenty-eight reflective markers^31^ were collected at 150 Hz to determine the kinematics of the lower extremity and pelvis segments using a three-dimensional motion capture system (Raptor-4; Motion Analysis Corporation, Santa Rosa, CA, USA). The ground reaction forces were collected at 300 Hz by five force platforms embedded in the laboratory floor. The raw markers and force signals were filtered with a low-pass Butterworth filter and cutoff frequencies of 10 Hz and 50 Hz, respectively. A 50 N vertical ground reaction force threshold was adopted to detect heel strike and toe off events. Further details can be found elsewhere^30^.

We calculated the mechanical powers of the ankle joint and foot segment by employing a six-degrees-of-freedom model of the ankle joint and foot. Further details of this method were previously described elsewhere^21–23^.

The ankle power was calculated as a sum of linear and angular power components, which in turn were calculated by the product of force and linear velocity and the torque and angular velocity, respectively:

$$ P_{ankle} = F_{ankle} \cdot \Delta V_{ankle} + M_{ankle} \cdot \omega_{ankle} $$

where $F_{ankle}$ and $M_{ankle}$ are the forces and torques acting on the ankle joint, $\Delta V_{ankle}$ is the difference between the ankle linear velocities computed in relation to the shank center of mass and the foot center of mass, and $\omega_{ankle}$ is the angular velocity of the ankle joint. The force and torque at the ankle were obtained from a three-dimensional inverse dynamics approach for the foot segment.

The foot power was also calculated as a sum of linear and angular power components:

$$ P_{foot} = GRF \cdot \left[ {V_{{cm_{foot} }} + \left( {\omega_{foot} \times R_{{cop/cm_{foot} }} } \right)} \right] + M_{free} \cdot \omega_{foot} $$

where $GRF$ is the ground reaction force, $V_{{cm_{foot} }}$ is the velocity of the center of mass of the foot segment, $\omega_{foot}$ is the foot angular velocity, $R_{{cop/cm_{foot} }}$ is the distance vector between the foot center of mass and the center of pressure, and $M_{free}$ is the free moment.

Finally, the total power of the ankle–foot system was calculated as the sum of the ankle and foot powers:

$$ P_{{{\text{sum}}}} = P_{ankle} + P_{foot} $$

The outcome variables in the study were the peak and work values obtained from the power data (*P*_*ankle*_, *P*_*foot*_, and *P*_*sum*_). The maximum values of the *P*_*ankle*_ and *P*_*sum*_ and the minimum values of *P*_*foot*_ during the propulsion phase of the stance were considered. The mechanical work was calculated as the integral over time of the *P*_*ankle*_ and *P*_*sum*_ data during the stance phase of walking. The *P*_*foot*_ work was calculated during the foot absorption phase as described elsewhere^28^. These variables were calculated from each stance and at each speed condition, and they were averaged across trials to represent the pattern of each subject at each condition. These values were then used for further analysis.

Linear mixed-effects regression models were employed to examine the effects of speed and age on each outcome variable. We fitted models that express the mean value across subjects of the outcome variables as a linear function of age and speed (separately, added, and multiplied) with a random intercept for each subject. Such models were necessary because each subject walked at three different speeds with respect to his/her own comfortable speed (and because each subject had a different comfortable speed, the speed values varied continuously across all subjects). Accordingly, the following equation was the most general model used:

$$ feature = \beta_{0} + \beta_{S} Speed + \beta_{A} AgeGroup + \beta_{SA} Speed \times AgeGroup + \left( {1{|}Subject} \right) + \epsilon $$

where *feature* is one of the nine outcome variables, β_0_ is the fixed intercept, Speed and AgeGroup are the possible fixed factors, β_S_, β_A_ and β_SA_ are the regression coefficients (slopes), (1|Subject) is a random intercept for each subject, and ϵ is a residual error.

The Speed factor was treated as a continuous variable (see Fig. 1 for the distribution of speeds). The AgeGroup factor was treated as a categorical factor (coded as Young = 0 and Older = 1); in this way, a positive regression coefficient for the AgeGroup factor would imply that age increases the values of the corresponding adjusted feature. We show scatter plots for all nine features versus speed and only the regression lines of models that yielded significant effects (Fig. 3).

As our intent for the use of linear regression was inferential, the outcome variables and the variable Speed were standardized (each variable was transformed to mean = 0 and standard deviation = 1). In this way, the standardized regression coefficients of the fixed factors can be used as an effect size measure, and a value not significantly different from zero (or with a confidence interval containing the value 0) supports the null hypothesis that there is no association between the predictor variable and the response variable. The following statistics of the fitted models are presented: standardized regression coefficients for Speed and AgeGroup, 95% confidence interval, and the p value for the *t* test of the null-hypothesis significance test of these regression coefficients. As a metric for goodness-of-fit and for comparing the significance of the models, we computed the coefficient of determination (R^2^), the log-likelihood (LLF) and the Akaike Information Criterion (AIC). The R^2^ was calculated between the actual response variable and the predicted variable considering only the corresponding fixed factors in the prediction. The regression computation was performed in two steps: first, we employed the maximum likelihood method to determine the LLF and AIC metrics of each model; then, we used the restricted maximum likelihood method to determine unbiased estimates of the model coefficients. A likelihood ratio test was used to evaluate the significance of an additional term in the model if the coefficient for that term was significantly different from zero. In simplified terms, a better fit (which better explains the predicted feature from the predicted factors) is the model with regression coefficients (slopes) different from zero, larger LLF and R^2^ values, and smaller AIC values. Both LLC and AIC values can only be used to compare the goodness of fit between models with different number of terms applied to the same data (there are no normative values for these metrics). In addition, the normality of residuals was visually checked with a scatter plot and quantified with Jarque–Bera test. A significance level of α = 0.05 was adopted. Finally, we provide custom-made software written in the Python language as a Jupyter notebook to replicate all the mentioned statistical analysis and data visualization (see Data availability).

## Data Availability

The dataset analyzed during the current study is available in the Figshare repository, https://doi.org/10.6084/m9.figshare.5722711.v4. The software codes developed to analyze the data is available in the GitHub repository, https://github.com/BMClab/papers/tree/master/age_speed_af_power.
